# Long‐term variation in environmental conditions influences host–parasite fitness

**DOI:** 10.1002/ece3.5321

**Published:** 2019-06-20

**Authors:** Karen Musgrave, Andrew W. Bartlow, Jeanne M. Fair

**Affiliations:** ^1^ Environmental Stewardship Los Alamos National Laboratory Los Alamos New Mexico; ^2^ Biosecurity and Public Health Los Alamos National Laboratory Los Alamos New Mexico

**Keywords:** blowflies, climate, environmental change, host–parasite interactions, nestlings, parasites

## Abstract

Long‐term data on host and parasite fitness are important for predicting how host–parasite interactions will be altered in an era of global change. Here, we use data collected from 1997 to 2013 to explore effects of changing environmental conditions on bird–blowfly interactions in northern New Mexico. The objectives of this study were to examine what climate variables influence blowfly prevalence and intensity and to determine whether blowflies and climate variables affect bird fledging success. We examined how temperature, precipitation, and drought affect two parasitic blowflies and their hosts, Western Bluebirds (*Sialia mexicana*) and Ash‐throated Flycatchers (*Myiarchus cinerascens*). We found that blowfly prevalence did not change over time. Blowfly intensity increased over time in bluebird nests, but not in flycatcher nests. More blowflies result in slightly higher fledging success in bluebirds, but not flycatchers. There was a significant interaction between blowflies and precipitation on bluebird fledging success. For flycatchers, there was a significant interaction between blowflies and temperature and between blowflies and drought severity on fledging success. Given that the southwest is projected to be hotter and have more frequent and prolonged droughts, we predict that flycatchers may be negatively impacted by blowflies if these trends continue. Future work should focus on investigating the role of both blowflies and climate on fledging success. Climate patterns may negatively impact host fitness through altered parasite pressure.

## 1 INTRODUCTION

Host–parasite interactions are among the most common and complex relationships among species. Parasites play an essential ecological role in populations and communities, exerting selective pressures on their hosts. Parasites influence host community and population structure, population densities, and coevolution (Dobson & Hudson, 1986). Understanding the ecology of host–parasite interactions is important for predicting how these interactions will be altered in an era of global change. Long‐term data on host fitness and parasite abundance in relation to environmental fluctuations are critical to make these predictions.

Increasing temperatures, changing precipitation patterns, and land use change are altering ecosystems (IPCC, 2014; Travis, 2003). Changing environmental conditions will have direct and indirect effects on host–parasite interactions (Lafferty, 1997; Lafferty & Kuris, 1999; Scharsack et al., 2016). Temperature has prominent effects on biological and physiological processes, such that host immune function and parasite abundance and virulence may be altered (Dawson, Hillen, & Whitworth, 2005; Scharsack et al., 2016). For example, high temperatures result in decreased survivorship of mosquitofish when infected with tapeworms (Granath & Esch, 1983). Additionally, warming arctic temperatures are increasing infection pressure of nematodes in muskoxen, most likely due to faster development rates (Kutz, Hoberg, Polley, & Jenkins, 2005). High temperatures coupled with other climate conditions may act against each other. Romo and Tylianakis (2013) found rates of aphid parasitism increased as temperature increased. During high temperatures and drought, parasitism decreased and aphid population growth increased (Romo & Tylianakis, 2013). Ectoparasites and parasites with free‐living life stages may be disproportionately affected by climate. These parasites are more exposed to environmental conditions than parasites that are always in a host (Brooks & Hoberg, 2007; Martinez & Merino, 2011).

Birds are often subject to parasitism by a variety of ectoparasites, including hematophagous larvae. The larvae of flies of the genus *Protocalliphora* and closely related *Trypocalliphora* (Diptera: Calliphoridae) are common parasites in bird nests and feed on the blood of nestlings (Eeva, Lehikoinen, & Nurmi, 1994; Heeb, Kolliker, & Richner, 2000; Hurtrez‐Boussès, Perret, Renaud, & Blondel, 1997; Marshall, 1981; Rothschild & Clay, 1952; Sabrosky, Bennett, & Whitworth, 1989). These parasites are often included under the same common name, avian blowflies. Female blowflies tend to oviposit from April through July in occupied nests and larvae likely hatch within 72 hr. Larvae feed on the nestlings and pupate within approximately 9–14 days and adults emerge around 14–21 days (Bennett & Whitworth, 1991). The difference between these genera is that *Trypocalliphora* is subcutaneous for the entire larval stage, while larvae of *Protocallihpora* feed intermittently on nestling blood (Sabrosky et al., 1989). As *Trypocalliphora* near maturity, they emerge from the skin and drop into the nest to pupate. Both emerge as adults to lay eggs in new nests (Sabrosky et al., 1989).

*Protocalliphora* spp. overwinter as adults; pupae cannot tolerate low winter temperatures (Bennett & Whitworth, 1991). There is evidence that activity of adult flies is somewhat temperature dependent. Bennett and Whitworth (1991) noted that adult blowflies were inactive at 7–10°C and rarely became active until a threshold ambient temperature of approximately 15.5°C was met. This temperature threshold differs between species. Furthermore, there is evidence that larval densities in occupied nests are somewhat temperature dependent. After experimentally manipulating temperature inside nests of tree swallows, Dawson et al. (2005) found that larval densities followed a curvilinear trend with temperature. Larval densities were low at both higher and lower temperatures but peaked at around 25°C. Pavel, Chutný, Petrusková, and Petrusek (2008) also found *Trypocalliphora* spp. only infested Meadow Pipit and Bluethroat nestlings during warmer summers with temperatures frequently exceeding 18°C.

The effects of blowflies on nestling survival and health are varied. There is evidence that suggests *Protocalliphora* spp. have minor to no effects on nestling mortality and that survival to fledging is generally not affected (DeSimone, Clotfelter, Black, & Knutie, 2018; Hannam, 2006; Howe, 1992; Simon et al., 2004). However, nestlings can exhibit decreased growth rates (Banbura et al., 2004; Cantarero, López‐Arrabé, Redondo, & Moreno, 2013; Gentes, Whitworth, Waldner, Fenton, & Smits, 2007), decreased metabolic rate, and decreased behaviors, such as preening and resting (Simon et al., 2005) when parasitized by *Protocalliphora* spp. Other studies have indicated *Protocalliphora* spp. can increase nestling mortality (Halstead, 1988; Miller & Fair, 1997; Shields & Crook, 1987). Effects of *Trypocalliphora* spp. are similarly varied. Different than *Protocalliphora* spp. that feed intermittently, *Trypocalliphora* spp. are subcutaneous, and cause damage to muscle and organ tissue (Sabrosky et al., 1989).

While there is still some discussion as to the breadth of effects *Protocalliphora* spp. have on nestlings, it seems that nestlings tolerate blowfly parasitism (DeSimone et al., 2018; Roby, Brink, & Wittmann, 1992; Simon et al., 2004). The mechanisms for tolerating parasitism by blowflies are not widely understood. There is some evidence that resistance is also an important defense strategy against blowflies (DeSimone et al., 2018). When female tree swallows had higher antibody responses, nests had fewer blowflies, and the nestlings had slightly higher antibody levels (DeSimone et al., 2018). Whether environmental conditions such as temperature, drought conditions, or precipitation have an influence on nestlings’ ability to tolerate or resist parasites has not been greatly explored. There is evidence that weather may play a role in a host's fitness costs of parasitism (De Lope, González, Pérez, & Møller, 1993; Merino & Potti, 1996; Møller et al., 2014) and that climate may affect a host's defenses against parasites (Møller et al., 2014). Further studies are needed to gain a better understanding of the variable and complex effects changing environmental conditions have on host–parasite interactions.

The effects of climatic changes on the complexity of host–parasite interactions are challenging to forecast due to the lack of data over a long time period. We use a 17‐year dataset of avian blowflies and cavity‐nesting birds in northern New Mexico, an area experiencing rapid environmental change. While the southwestern United States commonly experiences periods of drought, there is consensus that rising temperatures will result in more intense, frequent, and prolonged droughts (Garfin, Jardine, Merideth, Black, & LeRoy, 2013). Average daily temperatures for 2001–2010 were the highest in the southwest from 1901 to 2010, and average temperatures since 1950 have been warmer than any comparable length of time in at least 600 years (Garfin et al., 2013). Dramatic changes to southwestern ecosystems over the last few decades and climatic variation among years offers a study system to explore environmental conditions and host–parasite dynamics.

Most studies examine the effects of blowflies over one or two seasons, which makes accessing the effects of environmental conditions difficult. We examine survival to fledging over a much longer timescale by using data collected from 698 nests over a 17‐year period. We investigate how climate change affects the interactions between Western Bluebirds (*Sialia mexicana*) and *Protocalliphora occidentalis* as well as Ash‐throated Flycatchers (*Myiarchus cinerascens*) and *Trypocalliphora braueri*. Our main goal was to determine how the environment influences the interactions between blowflies and fledging success. The three specific objectives of this study were to (a) determine whether blowfly prevalence and intensity have changed over time from 1997 to 2013; (b) identify the environmental variables that influence changes in blowfly prevalence and intensity; and (c) identify the effects of both environmental variables and blowfly parasitism on fledging success of these species. Based on evidence that blowfly prevalence is somewhat temperature dependent, we hypothesize that blowfly prevalence and intensity have increased over time since there has been a general increase in temperature (Dawson et al., 2005; Pavel et al., 2008). Our second hypothesis is that fledging success of both species will be negatively affected by the combination of blowflies, higher temperatures, and drought (De Lope et al., 1993; Merino & Potti, 1996; Møller et al., 2014).

## 2 METHODS

### 2.1 Study area and field design

This study was conducted on Los Alamos National Laboratory (LANL) property in Los Alamos County in north central New Mexico. The Los Alamos National Laboratory is a multidisciplinary research institution but was established in 1943 as part of the Manhattan project to design atomic weapons. LANL has an ongoing assessment of potential site‐related contamination and ecological risks. All observed concentrations of constituents in areas at LANL that wildlife have access to are below the lowest observable adverse effect levels (LANL, 2018). Furthermore, reports from 2018 indicate that chemical concentrations detected in eggs of Western Bluebirds and Ash‐Throated Flycatchers were below levels associated with adverse effects (Gaukler, Hathcock, & Fair, 2018). The results of these studies indicate that it is unlikely historical contamination at the study site would influence the results of the present study.

The 111 km^2^ laboratory is situated on the Pajarito Plateau and consists of a series of relatively narrow mesas separated by deep, steep‐sided canyons that decrease in elevation from the Jemez Mountains down to the Rio Grande (2,400–1,650 m). Six major vegetation types are found in Los Alamos County along the west‐to‐east elevational gradient: subalpine grassland, spruce–fir forest, mixed conifer forest, ponderosa pine forest, piñon–juniper woodland, and juniper grasslands (Foxx, Pierce, Tierney, & Hansen, 1998). The nest boxes are located primarily in pinon–juniper woodlands and ponderosa pine forests.

**Figure 1:**
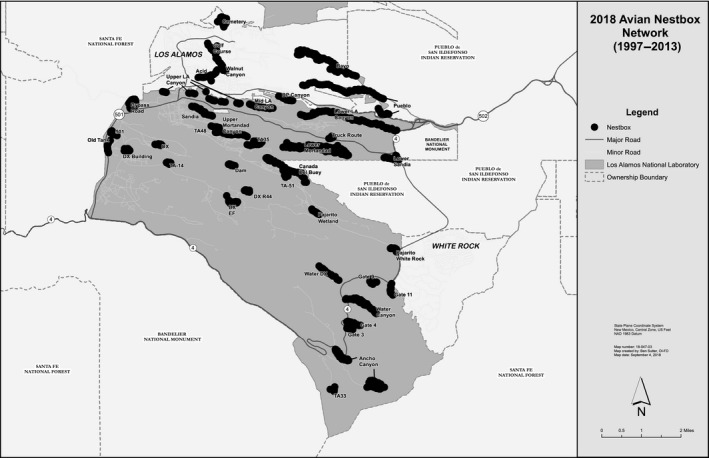
Map of the study area on the Pajarito Plateau in northern New Mexico where nest boxes were located

During the summer of 1997, we placed 438 nest boxes (Wild Birds Unlimited) across the Pajarito Plateau (Figure 1). Nest boxes were placed approximately two meters off the ground on ponderosa pine (*Pinus ponderosa*) and piñon pine (*Pinus edulis*) trees and spaced approximately 50–75 m apart. Boxes were placed in open ponderosa pine forest in canyons and piñon–juniper woodland on the plateau mesas. The elevations of individual nest boxes ranged from 1,892 to 2,212 m.

### 2.2 Study species

**Figure 2:**
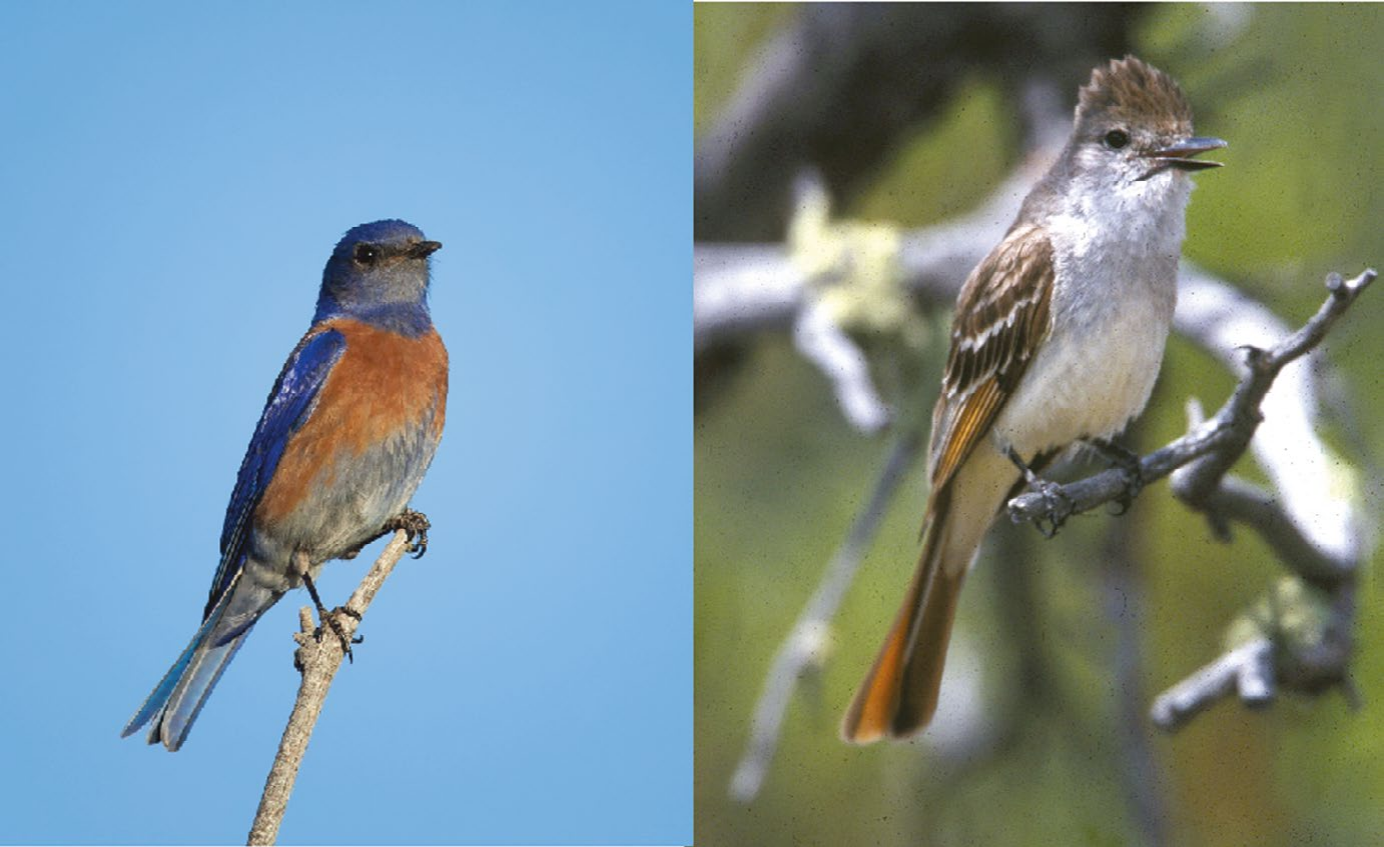
Adult Western Bluebird (*Sialia mexicana*) on the left and an adult Ash‐throated Flycatcher (*Myiarchus cinerascens*) on the right

Western Bluebirds (*Sialia mexicana*, hereafter bluebirds) and Ash‐throated Flycatchers (*Myiarchus cinerascens*, hereafter flycatchers) are two common migratory cavity‐nesting bird species that readily use nest boxes (Figure 2). Western Bluebirds are widely distributed, sexually dichromatic, and socially monogamous species. Ash‐throated Flycatchers are not as widely distributed or sexually dichromatic. Both species nest in secondary nest cavities in open woodlands and are insectivorous during the breeding season. With the exception of a few resident populations in the desert southwest and Baja California, the flycatcher population is almost entirely migratory. Bluebirds are short distant migrants, but largely resident, including resident populations in New Mexico and in the study area. These two species have similar life histories, with the exceptions that flycatchers grow more rapidly and fledge 4–5 days earlier than the bluebirds.

### 2.3 Nestling monitoring

Nest boxes were only monitored during the breeding season. Nest boxes were not visited during nonbreeding seasons. We visited nest boxes every two weeks beginning in May 1997 until August 2013. Nests with eggs were considered active and visited once a week. Nestling age and hatch date were based on the physical development of the nestlings (mass, tarsus length, and culmen) (Sanchez et al. in preparation). Each nestling was handled for less than five minutes in accordance with the Guidelines for the Use of Wild Birds in Research (Fair, Paul, & Jones, 2010). The Institutional Animal Care and Use Committees of both LANL and the University of Missouri‐St. Louis approved all protocols. Nests in which a nestling was missing before the expected fledge date (before 15 days) were presumed to be predated. Dead nestlings or unhatched eggs in the nest were removed from the nest and collected for laboratory analysis.

### 2.4 Parasite quantification

Nest boxes were cleaned of rodent nests and other material prior to spring nesting. We collected nest material from all nest boxes within one week of fledging and the nests were stored in plastic bags at room temperature. We removed adult flies the day they emerged from pupae, which was usually 2–5 days from collection. Nests were then stored in a freezer until sifted for pupae that did not emerge. Nests were searched thoroughly for pupae of *P. occidentalis* and *T. braueri*. Emergent adult insects were stored in methyl alcohol until identification. Other arthropod parts, seed food items, or unusual objects were removed and stored. All fly pupae were identified as *P. occidentalis* (in bluebird nests only) or *Trypocallihpora braueri* (in flycatcher nests only) by T. Whitworth in the early years of the study. Prevalence was defined as the number of nests that contained larvae, and parasite intensity was determined to be the number of larvae in only infested nests.

### 2.5 Climate data

All the environmental variables chosen were selected a priori to be biologically relevant factors for both blowflies and bird fledging success. These data were collected from the same centrally located weather station on the Pajarito Plateau (35.861°W 106.31°N, 2,263m.) (LANL, 2014). Temperature and precipitation data were collected from 1997 to 2013 for the summer breeding season (April‐August), from the weather station. Adult female flies tend to oviposit in nests containing very young nestlings (Sabrosky et al., 1989), and the nestling period lasts for 18–25 days for bluebirds and 13–17 days for flycatchers. Therefore, we chose mean monthly temperature, total monthly precipitation, and monthly Palmer Drought Severity Index (PDSI) values that corresponded with the month of the hatch date of the nest. PDSI was obtained using a Drought Atlas weather station location at 35.86°W and 106.321°N in Los Alamos, New Mexico (NDMC, 2018). PDSI is a meteorological drought index based on calculations of precipitation, temperature, and local available water content of the soil.

Because we were interested in determining how environmental variation influences blowfly prevalence and intensity in nests, we also used mean winter temperature and mean precipitation of the winter season preceding the breeding season (November–February). This was done to determine whether winter conditions influence blowfly prevalence and intensity during the breeding season since blowflies overwinter as adults (Sabrosky et al., 1989). We took the mean winter precipitation and mean winter temperature over the entire winter, from November through February because we wanted a snapshot of the environmental conditions during the preceding winter months.

### 2.6 Statistical analyses

All analyses were conducted using the statistical software R (version 3.4.0, R Core Team, 2018). Linear mixed models (LMM) and generalized linear mixed models (GLMM) were completed using the lme4 (version 1.1.17; (Bates, Mächler, Bolker, & Walker, 2015)) and lmerTest (version 3.0.1; (Kuznetsova, Brockhoff, & Christensen, 2017)) packages in R. Normality was assessed using the Lilliefors test using the nortest package (Gross &Ligges, 2015). Model selection was completed using the MuMIn package (Bartoń, 2018). All LMMs and GLMMs were done at the nest level, and the two species were analyzed separately. The number of blowflies in each nest was divided by the number of nestlings present in the nest to get a measure of parasites per nestling. These data were used when determining the best predictors of blowflies (i.e., a response variable) and when blowflies were predictor variables in models predicting fledging success. Blowfly intensity is the number of blowflies per nestling in only nests infested with blowflies. Only nests with complete data were used. The number of nests with complete data for bluebirds was 494, while there were 112 nests for flycatchers.

### 2.7 Changes in blowflies over time

To determine whether blowflies have increased or decreased over time, we used linear models and generalized linear models with year as the only predictor variable. Two separate tests were done to determine whether blowfly prevalence or blowfly intensity changed over time. These different tests allow us to understand more of the biology behind potential changes in blowfly populations over time. Generalized linear models with binomial distributions were used to determine whether blowfly prevalence changed over time. The second test was done on only infested nests, and the response variable was the number of blowflies per nestling. To make the model residuals more normal, blowflies per nestling was log transformed.

We also split up PDSI by drought category for each nest because we were specifically interested in how drought affects blowfly prevalence and intensity, even if PDSI was not a top predictor of blowflies. This is important because there are different severities of drought, and we wanted to do coarse comparisons among moist years, normal years, and three levels of drought. These categories were based on the following PDSI cutoffs: <−4 (extreme drought); −4 to −3 (severe drought), −3 to −2 (moderate drought), −2 to 2 (normal), 2–3 (moist); >3 (very moist). We plotted the prevalence in each category and mean blowfly intensity (±*SE*) in each category. To test for differences in prevalence, we used a generalized linear model with a binomial distribution. To test for differences in blowfly intensity among the drought categories, we ran a nonparametric Kruskal–Wallis test because an ANOVA produced residuals that were highly nonnormal. Transforming blowfly intensity did not improve normality of model residuals. Pairwise comparisons were analyzed using pairwise Mann–Whitney *U* tests with Bonferroni corrected p‐values, instead of comparing p‐values to a corrected significance level.

### 2.8 Model selection for blowflies

We used linear mixed models and GLMM to find the best set of variables to predict blowflies in the nests of each species. First, GLMM with binomial distributions allowed us to determine the best set of variables that influence whether nests are infested with blowflies (prevalence). Fixed effects in the models included the breeding season and winter environmental variables as well as hatch date of the nest. Year was the random effect in the models since several nests were sampled each year. If multicollinearity existed between two predictor variables (Pearson correlation coefficient, *r* > 0.7), the predictor variable that we predicted to have the biggest effect on the response variable was chosen. A global model was created with all environmental variables and hatch date, including interactions between environmental variables. Predictor variables were all standardized prior to model selection using the standardize function in the arm package (Gelman and Su, 2018). This was done to facilitate model convergence and interpretation of the parameter estimates (Grueber, Nakagawa, Laws, & Jamieson, 2011). The dredge function (MuMIn package) created all subsets of the global model, and models were ranked using AICc. The null intercept only model was included and ranked. Delta AICc values were used to find the top model or a subset of top models. A model was considered the best model if no other models were within 2 delta AICc values, and parameter estimates were reported from this model. In cases where models were within 2 delta AICc values, they were all considered top models. Model weights are presented and are the Akaike weights out of all the top models. Model averaging using the model.avg function (MuMIn package) was used to get the averaged parameter estimates and relative importance of each of the terms, including interaction terms. Conditional model‐averaged estimates are presented, which means that only models containing a given parameter are used to calculate the average.

Model selection was also done separately for parasite intensity (i.e., only those nests infested with blowflies). The response variable was number of blowflies per nestling for these linear mixed models. The residuals in initial models were highly nonnormal. Therefore, the blowflies were log transformed in models predicting blowfly intensity to make residuals more normal. The same model selection procedure using linear mixed models and the model averaging process was completed as described above.

### 2.9 Model selection for fledging success

Model selection was also used to determine the effects of blowfly parasitism and environmental variables on fledging success of these species. To determine the top model that predicts fledging success, we used GLMM with binomial distributions. Fledging success was determined by subtracting the number of nestlings that fledged from the number of nestlings that hatched. Thus, each nest had the number of successful fledges and unsuccessful fledges. A global model was created with all environmental variables, including interactions between each environmental variable and the number of blowflies per nestling. Predictor variables were all standardized prior to model selection. Interactions were included to test the hypothesis that environmental conditions would influence how blowflies affect fledging success. Year was included as a random effect in the mixed models to control for variation among years since multiple nests were sampled each year. Models were ranked using AICc and models within delta AIC values of 2 were averaged to get more precise parameter estimates, as described above.

Model selection was also done separately for blowfly intensity (i.e., only those nests infested with blowflies). Again, GLMM with binomial distributions were used. Interactions between each environmental variable and the number of blowflies per nestling were included. Model selection and model average were used to find the best set of predictor variables that predicted fledging success as described above.

## 3 RESULTS

From 1997 to 2013, 698 total active nests (clutch size of *n* > 0) were sorted and recorded. Of these active nests, 72% were parasitized by avian blowflies. There were 569 active bluebird nests with 75% of them parasitized. There were 129 active flycatcher nests with 61% parasitism. The number of blowflies per bluebird nestling in infested nests ranged from 0.2 to 55. The number of blowflies per flycatcher nestling ranged from 0.25 to 65. The number of nests each year for bluebirds and flycatchers is listed in Table 1 and Table 2, respectively. Also, listed are the number of nests infested with flies (prevalence) and summary information regarding fly intensity in nests each year. Here, mean intensity is the mean number of blowflies in infested nests; the number of nestlings has not been taken into account. Data from ninety‐two nests could not be used for the following modeling analyses due to missing data.

**Table 1:** Summary table for Western Bluebird nests from 1997 to 2013. Listed are the number of nests sampled each year, the number and percentage of nests infested (prevalence), the total number of flies, and the mean fly intensity and standard error

Year	*N* nests	*N* nests infested	Prevalence	Total flies	Mean fly intensity	*SE* fly intensity
1997	15	10	66.7	390	39.0	9.18
1998	38	26	68.4	556	21.4	3.99
1999	51	42	82.4	776	18.5	2.89
2000^a^	NA	NA	NA	NA	NA	NA
2001	12	10	83.3	243	24.3	8.00
2002	24	21	87.5	710	33.8	7.39
2003	39	31	79.5	812	14.6	4.08
2004	21	16	76.2	233	26.2	4.50
2005	43	34	79.1	1,416	41.6	5.85
2006	30	9	30.0	168	18.7	6.63
2007	60	45	75.0	1,525	33.9	6.98
2008	32	23	71.9	584	25.4	5.22
2009	57	38	66.7	925	24.3	3.96
2010	30	26	86.7	1,203	46.3	7.77
2011	46	42	91.3	2,363	56.3	6.64
2012	37	32	86.5	1,426	44.6	6.77
2013	34	23	67.6	596	25.9	4.30

a Due to a large wildfire during May 2000, no data were collected during the breeding season.

**Table 2:** Summary table for Ash‐throated Flycatcher nests from 1997 to 2013. Listed are the number of nests sampled each year, the number and percentage of nests infested (prevalence), the total number of flies, and the mean fly intensity and standard error

Year	*N* nests	*N* nests infested	Prevalence	Total Flies	Mean fly intensity	*SE* fly intensity
1997	13	5	38.5	87	17.4	6.49
1998	8	4	50.0	61	15.2	5.07
1999	14	8	57.1	132	16.5	6.48
2000^a^	NA	NA	NA	NA	NA	NA
2001	9	7	77.8	63	9.0	4.74
2002	15	12	80.0	252	21.0	6.28
2003	16	11	68.8	503	45.7	15.8
2004	8	6	75.0	213	35.5	31.9
2005	6	5	83.3	261	52.2	12.3
2006	6	0	0.0	0	0.0	0.0
2007	6	4	66.7	180	45.0	40.7
2008	4	3	75.0	24	8.0	4.36
2009	7	3	42.9	10	3.33	0.33
2010	7	5	71.4	358	71.6	28.6
2011	NA	NA	NA	NA	NA	NA
2012	5	5	100.0	172	34.4	16.9
2013	4	0	0	0	0	0

a Due to a large wildfire during May 2000, no data were collected during the breeding season.

### 3.1 Changes in blowflies over time

In bluebird nests, there was no significant change in blowfly prevalence over time for bluebirds (GLM: estimate = 0.013, *SE* = 0.022, *z* = 0.59, *p* = 0.56) or flycatchers (GLM: estimate = 0.012, *SE* = 0.042, *z* = 0.29, *p* = 0.77). When only infested nests were analyzed (intensity), the number of blowflies per nestling increased over time for bluebirds (LM: estimate = 0.041, *SE* = 0.013, *t* = 3.23, *p* = 0.001), but not for flycatchers (LM: estimate = 0.053, *SE* = 0.040, *t* = 1.34, *p* = 0.19).

**Figure 3:**
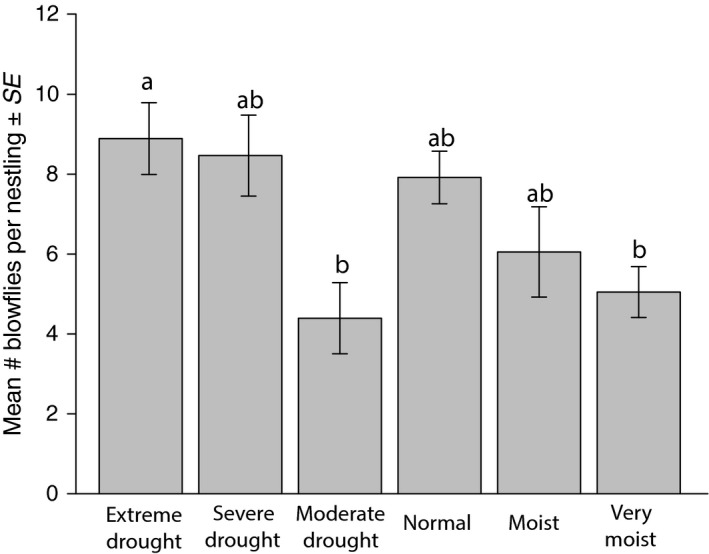
Blowfly intensity in bluebird nests according to the drought severity categories. There was a significant difference between the blowfly intensity in the six categories (Kruskal–Wallis: *χ* ^2^ = 17.82, *df* = 5, *p* = 0.003). Different letters denote significant differences based on pairwise Mann–Whitney *U* tests with Bonferroni corrections. Extreme drought was significantly different than moderate drought and very moist conditions

Among the six drought categories, there was no difference in blowfly prevalence for bluebirds (GLM: estimate = −0.028, *SE* = 0.068, *z* = −0.41, *p* = 0.68) or for flycatchers (GLM: estimate = −0.11, *SE* = 0.11, *z* = −0.96, *p* = 0.34). There was a significant difference between the blowfly intensity in the drought categories for bluebirds (Kruskal–Wallis: *χ* ^2^ = 17.82, *df* = 5, *p* = 0.003; Figure 3), but not for flycatchers (Kruskal–Wallis: *χ* ^2^ = 1.12, *df* = 5, *p* = 0.95). Two pairwise comparisons were significant for bluebird blowfly intensity: extreme drought and very moist (post hoc Mann–Whitney *U*: *p* = 0.004) and extreme drought and moderate drought (post hoc Mann–Whitney *U*: *p* = 0.02).

### 3.2 Model selection for blowflies

The best predictors of blowfly prevalence and intensity were determined using model selection based on ranking candidate models using AICc values. The results of the top models from the model selection for blowflies of both bluebirds and flycatchers are presented in Table 3. The model‐averaged parameters from these top models are shown in Table 4. For each response variable, models with environmental variables could not be distinguished from the intercept only null model. All intercept only models were just as likely to explain bluebird and flycatcher blowfly prevalence and intensity and ranked near the top according to delta AICc values (Table 3). After model averaging to get parameter estimate, none of the terms were significant (Table 4). This suggests that the environmental variables are not strong predictors for whether nests are infested with blowflies or how many blowflies infest nests.

**Table 3:** The top models (delta AICc < 2) for predicting blowfly prevalence and intensity for bluebirds and flycatchers. Monthly environmental variables (PDSI, mean temperature and total precipitation) are from the breeding season. Winter variables are means from November to February. Model weights were calculated from the set of top models

Species	Response variable	Parameters in model	AICc	Delta AICc	Model weight
Bluebirds	Blowfly prevalence	Hatch date	524.8	0.00	0.22
		Intercept only	525.4	0.55	0.17
		Mean winter temperature + hatch date	525.9	1.09	0.13
		Mean monthly temperature	526.1	1.33	0.11
		Total monthly precipitation	526.5	1.67	0.096
		Hatch date + winter monthly precipitation	526.5	1.69	0.095
		Mean winter temperature	526.6	1.74	0.093
		Hatch date + total monthly precipitation	526.8	1.96	0.083
Bluebirds	Blowfly intensity	Intercept only	1,184.6	0.00	0.43
		Total monthly precipitation	1,184.8	0.17	0.39
		PDSI	1,186.4	1.74	0.18
Flycatchers	Blowfly prevalence	Total monthly precipitation	151.9	0.00	0.16
		Intercept only	152.2	0.29	0.14
		Mean winter precipitation	152.2	0.29	0.13
		Mean monthly temperature + mean winter precipitation	153.1	1.24	0.084
		Mean winter precipitation + total monthly precipitation	153.1	1.25	0.083
		Mean monthly temperature + total monthly precipitation	153.1	1.25	0.083
		Hatch date	153.3	1.39	0.078
		Mean monthly temperature	153.6	1.72	0.066
		Hatch date + mean monthly temperature	153.7	1.80	0.063
		Mean winter temperature	153.8	1.92	0.059
		Hatch date + mean winter precipitation	153.8	1.95	0.059
Flycatchers	Blowfly intensity	Intercept only	248.0	0.00	0.25
		Mean monthly temperature	248.7	0.68	0.18
		Mean winter temperature + mean winter precipitation	248.8	0.75	0.17
		PDSI	249.4	1.30	0.13
		Total monthly precipitation	249.9	1.90	0.095
		Mean monthly temperature + mean winter temperature * mean winter precipitation	250.0	1.96	0.092
		Mean winter precipitation	250.0	1.98	0.091

**Table 4:** Model‐averaged parameter estimates from the top models (delta AICc < 2) predicting blowfly prevalence and blowfly intensity for bluebirds and flycatchers. The intercept only models were among the best models to explain blowfly intensity for both species. Monthly environmental variables (PDSI, mean temperature and total precipitation) are from the breeding season. Winter variables are means from November to February

Species	Response variable	Covariate	Relative variable importance	*B*	*SE*	*Z*	*p*
Bluebirds	Blowfly prevalence	Intercept		1.24	0.20	6.16	<0.001
		Hatch date	0.53	−0.37	0.23	1.57	0.12
		Mean winter temperature	0.22	−0.38	0.39	0.96	0.34
		Mean monthly temperature	0.18	−0.29	0.26	1.12	0.27
		Total monthly precipitation	0.11	−0.15	0.26	0.59	0.55
		Winter precipitation	0.10	0.23	0.40	0.59	0.55
Bluebirds	Blowfly intensity	Intercept only		1.44	0.11	13.14	<0.001
		Total precipitation	0.39	−0.28	0.14	2.039	0.04
		PDSI	0.18	−0.27	0.21	1.28	0.20
Flycatchers	Blowfly prevalence	Intercept		0.43	0.27	1.60	0.11
		Total monthly precipitation	0.32	−0.69	0.48	1.42	0.16
		Mean winter precipitation	0.36	0.75	0.61	1.22	0.22
		Mean monthly temperature	0.30	0.53	0.50	1.05	0.30
		Hatch date	0.20	−0.48	0.47	1.01	0.31
		Mean winter temperature	0.06	−0.39	0.58	0.67	0.50
Flycatchers	Blowfly intensity	Intercept		1.44	0.24	5.89	<0.001
		Mean monthly temperature	0.27	0.48	0.40	1.19	0.23
		Mean winter temperature	0.26	0.15	0.50	0.288	0.77
		Mean winter precipitation	0.35	0.03	0.51	0.062	0.95
		Mean winter temperature * mean winter precipitation	0.26	1.81	0.98	1.82	0.069
		PDSI	0.13	−0.39	0.42	0.91	0.36
		Total monthly precipitation	0.10	−0.28	0.37	0.77	0.45

### 3.3 Model selection for fledging success

**Figure 4:**
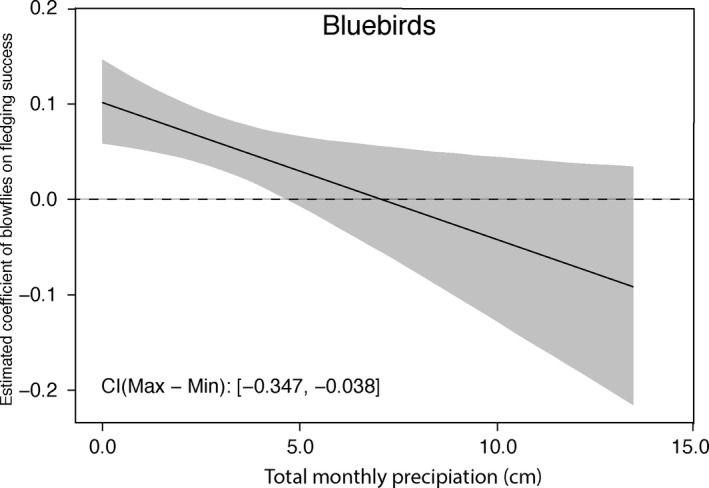
The estimated coefficient of the number of blowflies per nestling on bluebird nestling fledging success according to the total monthly precipitation. More precipitation results in a decrease in the effect of blowflies on fledging success. The effect of blowflies on fledging success becomes negative around 7 cm of precipitation

Next, we determined the best set of environmental variables for bluebird and flycatcher fledging success. For bluebird fledging success using all nests (i.e., those infested with blowflies *and* those not infested), all three of the top three models included the interaction between blowflies per nestling and total monthly precipitation (Table 5). Blowflies per nestling, total monthly precipitation, and the interaction between them were each significant after model averaging and each had a relative importance of 1.00 (Table 6). The same models were also the best models explaining bluebird fledging success on only nests that were infested with blowflies (Table 5). These models suggest that the effect of the number of blowflies per nestling on fledging success varies according to environmental conditions, specifically the amount of precipitation during the month the nest hatched. According to the models, as the number of blowflies per nestling increases, the higher the fledging success of bluebirds. The significant negative interaction with total monthly precipitation means that higher monthly precipitation decreases the positive effect of blowflies fledging success (Figure 4). Mean monthly temperature and PDSI were also variables in the top three models. However, these variables were not significant after model averaging and were determined to have the least relative importance of all the variables (Table 6).

**Table 5:** The top models (delta AICc < 2) for predicting fledging success for bluebirds and flycatchers. Fledging success using all nests and those using only infested nests were modeled separately. Monthly environmental variables (PDSI, mean temperature and total precipitation) are from the breeding season. Model weights were calculated from the set of top models

Species	Response variable	Parameters in model	AICc	Delta AICc	Model weight
Bluebirds	Fledging success	Blowflies/nestling * total monthly precipitation	1,137.3	0.00	0.38
		Blowflies/nestling * total monthly precipitation + mean winter temperature	1,138.7	1.42	0.19
		Blowflies/nestling * total monthly precipitation + mean monthly temperature	1,139.2	1.95	0.15
		Blowflies/nestling * total monthly precipitation + PDSI	1,139.3	1.98	0.14
Bluebirds	Fledging success of only infested nests	Blowflies/nestling * total monthly precipitation	843.1	0.00	0.51
		Blowflies/nestling * total monthly precipitation + mean monthly temperature	844.4	1.31	0.27
		Blowflies/nestling * total monthly precipitation + PDSI	844.7	1.66	0.22
Flycatchers	Fledging success	Blowflies/nestling * mean monthly temperature	160.9	0.00	0.29
		Blowflies/nestling * PDSI + mean monthly temperature	161.9	0.97	0.18
		Mean monthly temperature	162.1	1.14	0.17
		Blowflies/nestling + mean monthly temperature	162.6	1.63	0.13
		Blowflies/nestling + mean monthly temperature + mean winter temperature	162.8	1.81	0.12
		Blowflies/nestling * mean monthly temperature + PDSI	162.9	1.98	0.11
Flycatchers	Fledging success of only infested nests	Mean monthly temperature + mean winter temperature	93.3	0.00	0.28
		Mean monthly temperature + mean winter temperature + blowflies/nestling	93.5	0.18	0.26
		Mean monthly temperature + mean winter temperature + total monthly precipitation	94.8	1.48	0.14
		Mean monthly temperature + blowflies/nestling	95.2	1.91	0.11
		Mean monthly temperature + mean winter temperature + blowflies/nestling + total monthly precipitation	95.3	1.96	0.11
		Mean monthly temperature * blowflies/nestling + mean winter temperature	95.3	1.98	0.11

**Table 6:** Model‐averaged parameter estimates from the top models (delta AICc < 2) predicting fledging success for bluebirds and flycatchers. Monthly environmental variables (PDSI, mean temperature and total precipitation) are from the breeding season. Winter variables are means from November to February

Species	Response variable	Covariate	Relative variable importance	*B*	*SE*	*Z*	*p*
Bluebirds	Fledging success	Intercept		2.01	0.17	11.54	<0.001
		Blowflies/nestling	1.00	0.84	0.20	4.15	<0.001
		Total monthly precipitation	1.00	−0.43	0.17	2.52	0.01
		Blowflies/nestling * total monthly precipitation	1.00	−1.10	0.45	2.45	0.01
		Mean winter precipitation	0.22	−0.27	0.34	0.80	0.43
		Mean monthly temperature	0.17	−0.052	0.16	0.32	0.75
		PDSI	0.17	0.09	0.35	0.26	0.79
Bluebirds	Fledging success of only infested nests	Intercept		2.04	0.17	11.99	<0.001
		Blowflies/nestling	1.00	1.08	0.244	4.43	<0.001
		Total monthly precipitation	1.00	−0.43	0.202	2.12	0.03
		Blowflies/nestling * total monthly precipitation	1.00	−1.34	0.532	2.53	0.01
		Mean monthly temperature	0.27	0.16	0.186	0.87	0.38
		PDSI	0.22	−0.21	0.336	0.63	0.53
Flycatchers	Fledging success	Intercept		2.94	0.42	6.99	<0.001
		Blowflies/nestling	0.83	1.71	1.27	1.34	0.18
		Mean monthly temperature	1.00	−1.37	0.66	2.05	0.04
		Blowflies/nestling * mean monthly temperature	0.52	−3.43	1.94	1.75	0.08
		Blowflies/nestling * PDSI	0.18	5.31	3.17	1.66	0.10
		PDSI	0.29	0.17	1.01	0.17	0.87
		Mean winter temperature	0.12	−0.34	0.55	0.61	0.55
Flycatchers	Fledging success of only infested nests	Intercept		3.00	0.46	6.38	<0.001
		Mean monthly temperature	1.00	−2.07	0.73	2.79	<0.01
		Mean winter temperature	0.89	−1.58	0.81	1.91	0.056
		Blowflies/nestling	0.58	1.36	1.10	1.22	0.22
		Total monthly precipitation	0.24	−0.58	0.67	0.84	0.40
		Blowflies/nestling * mean monthly temperature	0.11	−1.76	2.34	0.74	0.46

**Figure 5:**
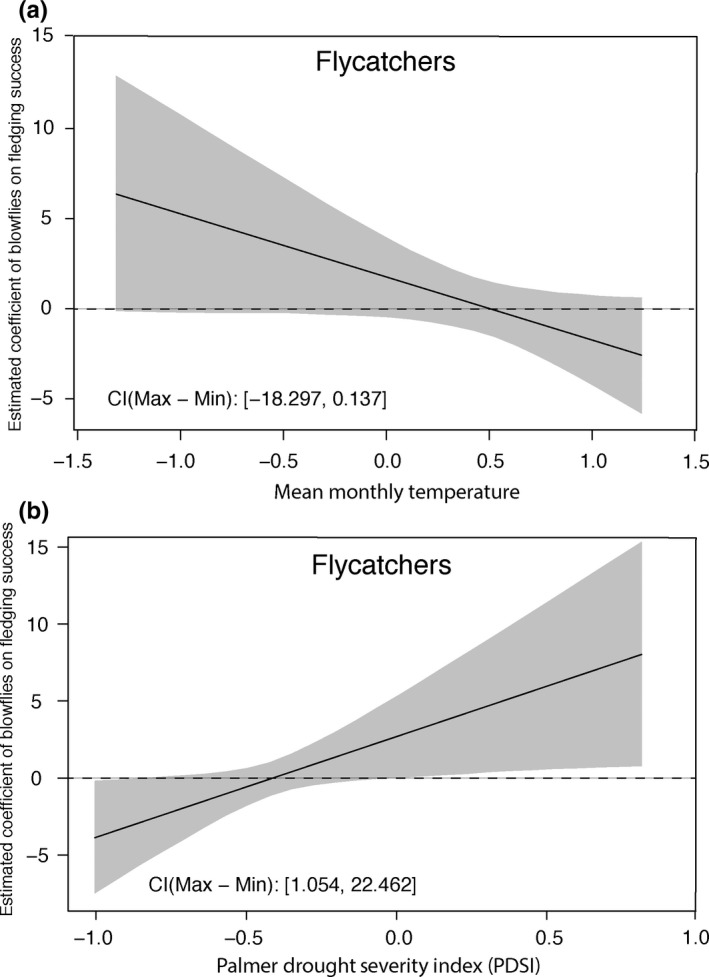
The estimated coefficient of the number of blowflies per nestling on flycatcher nestling fledging success according to (a) mean monthly temperature and (b) Palmer Drought Severity Index (PDSI). The values on the x‐axes were standardized during the model selection process (see methods). (a) When temperatures are high, the effect of blowflies on fledging success is more negative compared to low temperatures. (b) During periods of high PDSI values (i.e., wetter conditions), blowflies have a positive effect of flycatcher fledging success compared to periods of low PDSI values (i.e., drought)

For flycatchers, the top models for fledging success did not include the interaction between blowflies per nestling and total monthly precipitation (Table 5). Using all nests (i.e., those infested *and* not infested), the top models contained other variables, such as mean monthly temperature and PDSI (Table 5). There were two interactions important in the top models for flycatcher fledging success for all nests. The interaction between the number of blowflies per nestling and mean temperature was the top ranked model for fledging success of all nests (Table 5). With higher mean monthly temperature, the positive effect of blowflies on fledging success decreases (Figure 5a). The second‐best model had the interaction between the number of blowflies per nestling and PDSI (Table 5). As the drought severity lessened (i.e., the environment became wetter), the positive effect of blowflies on fledging success increased (Figure 5b). For flycatcher fledging of only infested nests, mean monthly temperature and mean winter temperature were in most of the top models and were the variables with the highest relative importance.

## 4 DISCUSSION

The main goal of this study was to use a long‐term dataset to determine how environmental conditions influence the interactions between blowflies and fledging success through three specific objectives. We determined whether blowfly prevalence and intensity changed over time from 1997 to 2013, identified environmental variables that influence blowfly prevalence and intensity, and identified effects of both environmental variables and blowfly parasitism on fledging success.

We did not find a detrimental effect of blowflies on bluebird and flycatcher fledging success similar to other studies (Hannam, 2006; Simon et al., 2004; Wittmann & Beason, 1992). Contrary to predictions and other studies, blowflies had a positive effect on bluebird fledging success. These results are consistent with other findings in the same population, but at the nestling level. Bluebird and flycatcher nestlings have recently been shown to be heavier and have longer tail and tarsus lengths when parasitized by blowflies, although the effects are small (J. M. Herman, in prep.). This may be due to compensatory feeding behaviors or other strategies such as accelerating growth toward the end of the nestling period (Killpack, Tie, & Karasov, 2014). Delaying fledging can also compensate for the negative effects of parasitism, (Hannam, 2006; Simon et al., 2004) although this was not analyzed in this study. Flycatcher nests in our study had a fairly high rate of parasitism (61% of nests were parasitized) and had no effect, positive or negative, on fledging success.

These results align with other studies that suggest nestlings generally tolerate parasitism by blowflies (DeSimone et al., 2018). Future studies could benefit from other measures of fledging health, such as immunocompetence and fitness postfledging. We cannot rule out the possibility that host defensive strategies decrease parasites in the nest (DeSimone et al., 2018), since we do not have data on nestling and adult health and condition. However, studies have yet to provide convincing evidence that this occurs. Our study examines the relationships among three key players (i.e., the environment, parasites, and hosts) in the direction of environment–parasites–hosts. We do not examine these relationships in the direction of environment–hosts–parasites. Host defense in these systems is needed to get a better understanding of the entire process.

One would predict that compensatory strategies would be unsuccessful when other environmental stressors exist (Dufva & Allander, 1996; De Lope et al., 1993; Pavel et al., 2008), such as a lack of resources due to drought conditions. This may be the case, however, our results suggest there is considerable variability between species and environmental conditions. For bluebirds, the interaction of precipitation and blowflies was the greatest predictor of fledging success. The interaction between the number of blowflies per nestling and total monthly precipitation was included in all of the top models predicting bluebird fledging success. The estimate of this interaction term is negative, which means that as the amount of precipitation increases, the positive effect (greater fledging success within nests) of blowflies on fledging success decreases. Therefore, high precipitation could result in negative impacts of blowflies on bluebird fledging. These results are similar to those in Meadow Pipits (*Anthus pratensis*), in which the combination of blowfly infection and high rainfall reduced nestling survival (Pavel et al., 2008). Similarly, other studies have suggested adverse weather (heavy rainfall) can negatively affect a host's ability to defend against parasites (Howe, 1992). Total monthly precipitation by itself was a significant predictor of fledging success, similar to other studies (Radford, Woodburn, & Morecroft, 2001; Shiao, Chuang, Yuan, & Wang, 2015). However, our results suggest that the interaction of precipitation and blowflies was a greater predictor of fledging success than precipitation alone.

Interactions between the number of blowflies per flycatcher nestling and environmental variables were also important in determining flycatcher fledging success, particularly mean breeding season temperature and PDSI. Higher temperatures resulted in the positive effect of blowflies on fledging success to decrease. Drier conditions (low PDSI values) were associated with negative effects of blowflies (decreased fledging success) than in moist conditions. Flycatchers may be more stressed in drought conditions compared to bluebirds and may not be able to tolerate blowflies. In addition, this blowfly species (*T. braueri*) burrows into the skin of its host, causing subcutaneous myiasis, further increasing the negative consequences of infection. Even though these interactions were not significant, they were in the top models explaining flycatcher fitness. Sample sizes may have been too low to find significant interactions in this study. We suggest investigating these interactions in the future to better understand the role of drought and temperature on blowfly virulence and host–parasite defense.

Unlike bluebirds, the top models for flycatchers included different variables when only infested nests were included versus when all nests were included. Flycatcher nests that were infested with blowflies were affected by mean temperature of the hatch month and mean temperature from November to February. High temperatures in the hatch month and high winter temperatures resulted in reduced fledging success when nests were infested. The number of blowflies per nestling was present in the top models but was not an important variable in predicting fledging success. When infested with blowflies, flycatcher fledging may be more dependent on conditions prior to the breeding season than bluebirds. For both analyses for bluebirds (i.e., all nests and only infested nests), the interaction between number of blowflies and total monthly precipitation during the breeding season was among the best variables predicting fledging success.

We found a significant increase in blowfly intensity from 1997 to 2013 for bluebirds. However, blowfly prevalence did not increase over time for either species, which means blowflies are not infesting more nests. For bluebirds, there are more blowflies per nestling, but not for flycatchers, suggesting that bluebird nestlings are being subjected to higher parasitism by blowflies now than they were 17 years ago. Flycatchers seem to have been subjected to the same level of parasitism over the study period. If the trend of greater blowflies over time continues, bluebirds may not be able to tolerate high levels of blowfly parasitism, especially in periods of high rainfall. This increase in parasite load in bluebird nests was not directly correlated with temperature, precipitation, or drought conditions. However, a change in parasite load over time warrants additional studies. Future work should determine whether a threshold level of parasitism exists, over which fledging is greatly reduced.

Blowfly prevalence and intensity were not affected by environmental variables as has been shown in other studies (Dawson et al., 2005; Merino & Potti, 1996; Pavel et al., 2008; Poulin, 2006). For both blowflies in bluebird and flycatcher nests, the null intercept only model was just as likely from the top models from all the models tested, including those with interactions. This means that the model with no environmental variables was just as good as models including environmental variables. Mean winter temperature and mean winter precipitation were not important in predicting blowfly prevalence and intensity during the breeding season, contrary to our hypothesis. Even though blowflies overwinter as adults, survival may not be affected by cold temperatures and high amounts of precipitation (Bennett & Whitworth, 1991). While cold temperatures seem to affect activity levels of adult blowflies (Bennett & Whitworth, 1991), our results indicate overwintering environmental conditions do not necessarily affect breeding season prevalence or intensity. Future work should determine what combination of environmental variables is important for blowfly populations. Even though environmental variables examined here had negligible effects on blowflies, there are likely certain combinations of conditions that are favorable for blowflies (as shown in Dawson et al., 2005). Larger sample sizes and additional years of data may be required to identify these conditions in the context of environmental change.

Blowfly prevalence and intensity in flycatcher nests did not differ among the six drought categories, but blowfly intensity of bluebirds did differ among categories. The extreme drought category had significantly more blowflies than the moderate drought and very moist categories. During periods of drought, a host may be more susceptible to parasites (Møller, Erritzøe, & Saino, 2003; Møller, Martín‐Vivaldi, Merino, & J. Soler, 2006; Plischke, Quillfeldt, Lubjuhn, Merino, & Masello, 2010). It is possible that the parasite load in bluebird nests increased under extreme drought conditions because host condition was weakened. Other host–parasite systems such as prairie dogs and fleas in New Mexico have shown similar results (Eads, Biggins, Long, Gage, & Antolin, 2016). However, we were unable to collect host condition data for this study. Future work should focus on examining the effects of extreme drought on host condition and susceptibility to parasitism.

Drought conditions and higher temperatures are predicted to continue in the southwest due to climate change (MacDonald, 2010). Drought affects birds at the community level, such as decreases in species abundance, richness, and composition (Albright et al., 2010; Fair, Hathcock, & Bartlow, 2018). Drought also affects individuals. A lack of resources, as a result of a disturbance such as drought, often results in suppressed immune system, low weight, and lower reproductive success (Alonso‐Alvarez & Tella, 2001). For instance, nestling bluebirds and flycatchers on the Pajarito Plateau experience a decrease in cell‐mediated immune responsiveness during unusually dry weather conditions (Fair & Whitaker, 2008). Given that the southwest is projected to be hotter and have more frequent and prolonged droughts (Cayan et al., 2013), we predict that flycatchers may be more negatively impacted by blowflies than bluebirds. Future work should focus on investigating the role of both blowflies and climate on fledging success between species.

Altered parasite pressure through new climate patterns and increased variation of environmental conditions may be an unforeseen consequence of climate change for many species. Relatively stable host–parasite systems may become unstable, resulting in population declines. Our results generally concur with previous studies that nestlings tolerate parasitism by blowflies. However, our results also indicate there may be an interaction between climate conditions and parasitism on fledging success that could ultimately lead to a decrease in fledging success. Our results also suggest that environmental variables *and* blowflies were better predictors of fledging success than just blowflies or environmental variables alone. These results provide opportunities for future studies to explore these relationships in greater depth. Using fledging success as the only indicator of tolerance to parasitism was a limitation in this study. Future studies would benefit from using nestling condition to examine in depth how the host condition may influence parasite prevalence and intensity.

## CONFLICT OF INTEREST

We declare no competing interests.

## AUTHOR CONTRIBUTIONS

JMF conceived the study, collected the data, and edited the manuscript; AWB and KSM analyzed the data and wrote the manuscript.

## Data Availability

Research data are not shared.
